# Fungal Diversity in Field Mold-Damaged Soybean Fruits and Pathogenicity Identification Based on High-Throughput rDNA Sequencing

**DOI:** 10.3389/fmicb.2017.00779

**Published:** 2017-05-03

**Authors:** Jiang Liu, Jun-cai Deng, Cai-qiong Yang, Ni Huang, Xiao-li Chang, Jing Zhang, Feng Yang, Wei-guo Liu, Xiao-chun Wang, Tai-wen Yong, Jun-bo Du, Kai Shu, Wen-yu Yang

**Affiliations:** ^1^Key Laboratory of Crop Ecophysiology and Farming System in Southwest, Ministry of AgricultureChengdu, China; ^2^Institute of Ecological Agriculture, Sichuan Agricultural UniversityChengdu, China; ^3^Sichuan Engineering Research Center for Crop Strip Intercropping SystemChengdu, China

**Keywords:** soybean, field mold, fungal diversity, pathogenicity identification, rDNA ITS

## Abstract

Continuous rain and an abnormally wet climate during harvest can easily lead to soybean plants being damaged by field mold (FM), which can reduce seed yield and quality. However, to date, the underlying pathogen and its resistance mechanism have remained unclear. The objective of the present study was to investigate the fungal diversity of various soybean varieties and to identify and confirm the FM pathogenic fungi. A total of 62,382 fungal ITS1 sequences clustered into 164 operational taxonomic units (OTUs) with 97% sequence similarity; 69 taxa were recovered from the samples by internal transcribed spacer (ITS) region sequencing. The fungal community compositions differed among the tested soybeans, with 42 OTUs being amplified from all varieties. The quadratic relationships between fungal diversity and organ-specific mildew indexes were analyzed, confirming that mildew on soybean pods can mitigate FM damage to the seeds. In addition, four potentially pathogenic fungi were isolated from FM-damaged soybean fruits; morphological and molecular identification confirmed these fungi as *Aspergillus flavus, A. niger, Fusarium moniliforme*, and *Penicillium chrysogenum*. Further re-inoculation experiments demonstrated that *F. moniliforme* is dominant among these FM pathogenic fungi. These results lay the foundation for future studies on mitigating or preventing FM damage to soybean.

## Introduction

Soybean (*Glycine max* L. Merr.) is an important oilseed and protein crop, and its fruits are highly prone to mildew-induced deterioration due to their high levels of protein and oil (Vollmann et al., 2000). In Southwest China, where karst topography is common, the amount of precipitation during autumn accounts for approximately one-quarter of the annual rainfall (Liu et al., 2015). In these areas, to coordinate with the autumn rainfall occurring over the western part of the country, soybean is sown at the beginning of June and harvested at the end of October (Yang et al., 2014). However, during this wet season, the exposure of unharvested soybeans to prolonged and continuous rainfall promotes the growth of field mildew fungi, which propagate rapidly and reduce soybean yield and quality (Deng et al., 2015b). This pre-harvest soybean deterioration due to fungal infection in the field is termed field mold (FM) and occurs in soybean production areas worldwide. For example, in the first systematic report of FM, abnormally warm and humid weather from September 18 to October 7, 1986, delayed soybean harvest in five US states (Jacobsen et al., 1995).

Field mold infection during seed development both alters the primary metabolites of soybean fruits, including oils, proteins and fatty acids, and changes the biosynthesis of secondary metabolites, such as isoflavone, tocopherol, and phenolic acid (Gutierrez-Gonzalez et al., 2010; Chennupati et al., 2011). Although in practice, FM has been a major obstacle to soybean production in Southwest China, there is little information about the pathogenic fungi and host response mechanism involved. Thus, it is urgently necessary to identify the FM pathogenic fungi in order to expand research on the mechanisms of resistance and the development of preventive measures.

In this study, we used Illumina high-throughput sequencing of the nuclear ribosomal internal transcribed spacer-1 (ITS1) to investigate the fungal diversity of four different soybean varieties with different FM resistances. Here, we report for the first time the isolation, purification and identification of FM fungi using morphological and molecular methods. The high host specificity of FM fungal diversity among different soybean varieties is also discussed.

## Materials and Methods

### Experimental Design and Sampling

This study used four soybean varieties: two FM-susceptible varieties, “ND12” and “GX1,” which have a yellow seed coat and are conventional cultivars in Southwestern China, and two highly resistant germplasms, “C103” and “2162,” which have a black seed coat and are grown in Sichuan Province. The soybean plants were grown in pots in the experimental field of Sichuan Agricultural University in Ya’an, China (103°00′E, 30°08′N). Six seeds were sown per pot, and 2 weeks after sowing, the seedlings were thinned to three plants per pot. The soybean plants were transferred from the field to a solar greenhouse approximately 5 days before growth stage R7 (beginning maturity). Plants in the greenhouse were exposed to day/night temperatures of 21/13°C and humidity of 85–100% for 7 days during the remainder of the seed development and maturation period, according to Keigley and Mullen (1986) with some modifications. The soybean fruits (seeds and pods) were harvested at stage R8 (full maturity), when mildew covered the plants. Three biological replicates of each soybean variety were evaluated. The fruits from the middle portion of the plants were collected and immediately stored in sterile plastic tubs reported to avoid aerial contamination. The tubs were sealed and stored at -80°C until further analysis.

### Organ-Specific Mildew Survey

The mold levels of the seeds and pods of these four varieties were determined by estimating the percentage of the seed/pod area that was mildewed. The details of this method were described in our previous study (Liu et al., 2016). Briefly, mildew rate= 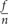 × 100 (*f*, mold seed/pod levels; *n*, number of surveys). Mildew index = 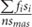 × 100 (*f_i_*, mold seed/pod levels of various grades; *s_i_*, corresponding mold grades; *n*, number of surveys; *s_mas_*, top mold grade). These mildew indexes were subjected to regression analyses using all the experimental data to construct mathematical models using Microsoft Excel 2016 and SPSS software.

### DNA Extraction and Illumina Sequencing

Samples were collected from the same five surfaces of soybean fruits from three randomly chosen sterile plastic tubs. Total genomic DNA from 5 g of soybean samples was extracted directly using the FastDNA^®^ spin kit (MP Bio, Santa Ana, CA, USA) according to the manufacturer’s instructions. The DNA was diluted to 1 ng/μL using sterile water and amplified using the primers ITS1F (5′-CTTGGTCATTTAGAGGAAGTAA-3′) and ITS2 (5′-GCTGCGTTCTTCATCGATGC-3′), which target the ITS1 region of the fungal rRNA gene (Krumins et al., 2009). The PCR amplifications were conducted using TransGen AP221-02 (TransStart Fast Pfu DNA Polymerase) and an ABI GeneAmp^®^ 9700. Amplification was assessed by analyzing 3 μL of product on 2% agarose gels. Samples with an intense band between 100 and 500 bp were chosen for further experiments. Sequencing libraries were generated using the NEB Next^®^ Ultra^TM^ DNA Library Prep Kit for Illumina (NEB, USA) following the manufacturer’s recommendations, and index codes were added. The library quality was assessed using the QuantiFluor^TM^-ST Blue fluorescence quantitative system (Promega), and the library was sequenced using the Illumina MiSeq platform at Majorbio Bio-Pharm Technology Co., Ltd., Shanghai, China, according to standard protocols.

### OTU Cluster and Diversity Analysis

Operational taxonomic unit (OTU) clustering, species annotation and diversity analysis were conducted according to Zhang et al. (2016). OTUs were assigned using USEARCH (version 7.1^1^) with a threshold of 97% pairwise identity (Zhang et al., 2016). The normalized OTU abundance information was utilized for alpha (single sample analysis) and beta (multivariate sample community) diversity analyses. Metrics of community richness included the Chao1 and ACE estimators. Community diversity was assessed using the Shannon and Simpson indices. Coverage, indicating sequencing depth, was evaluated using Mothur version v.1.30.1 (Schloss et al., 2011). For beta diversity, principal component analysis (PCA) was applied for the multivariate examination of OTUs (Wang et al., 2012), and Bray-Curtis dissimilarity (Srinivasan et al., 2012) was calculated using the R package (Jami et al., 2013).

### Fungal Isolation and Morphological Identification

Fungi on FM-damaged soybean fruits were isolated using the suspension plating method (Zhou et al., 2014). In brief, 2 g of each sample was added to 50 mL of sterilized water, and the mixture was rotated for 1 day to produce a 10^-1^ slurry (w/v); the suspension was then diluted to final concentrations of 10^-2^, 10^-3^, 10^-4^, and 10^-5^. A 0.1-mL aliquot of the 10^-3^, 10^-4^, and 10^-5^ suspensions was placed in a 90-mm-diameter Petri dish containing 20 mL of Martin’s rose bengal agar (henceforth Martin medium) with streptomycin (at approximately 40°C) and spread evenly using sterile coated rods. Sterilized water was used as the control. The plates were kept in the dark at 37°C for 1–2 days. Single fungal colonies with morphological differences were selected from the mixed populations, sub-cultured and purified using the streak plate method (Bondici et al., 2013). Distinct colonies, based on morphological type and color, were selected for isolation and subsequent taxonomic and molecular identification. All isolates were sub-cultured and initially grouped into morphotypes based on morphological characters. The fungi were first identified to the genus or species level based on the morphological characteristics reported in the original taxonomic literature (Chakrabarti and Ghosal, 1989; Amoah et al., 1995) and then further identified based on ITS sequence comparison.

### Pathogenicity Identification

To confirm the pathogenicity of the isolated fungi, preliminary pathogenicity testing of the fungal isolates was performed according to Koch’s postulates. Each isolated fungus was first grown on Martin medium in the dark at 28°C for 7 days until reaching sporulation. Each fungus was mixed with sterilized water to produce a fungal suspension of 2 × 10^6^ spores/mL. Healthy seeds of the FM-susceptible variety “ND12” with no defects were infected with the fungal suspension in 5-cm Petri dishes containing moistened filter paper to encourage spore germination on the seed surface. Sterilized water was used as a blank control. All treatments were performed in triplicate. The Petri dishes were sealed with film to maintain a suitable relative humidity (98∼100%) and incubated at 25–28°C in darkness for 7 days. The samples were inspected daily until the 7th day, at which point the levels of mold on the infected seeds were determined according to the method of Li et al. (2005) with slight modifications. These procedures were described in our previous study (Deng et al., 2015b). The pathogenicity of each fungus was confirmed using a previously reported fungal recovery procedure (Ali-Shtayeh et al., 2003).

### PCR Amplification and Sequencing Analysis

The isolated pure pathogenic fungi with different morphotypes were grown on Martin medium in the dark at 28°C for 5 days until reaching sporulation. The mycelia were placed in a sterilized mortar, immediately frozen in liquid nitrogen, and ground into a dry powder for further analysis. Genomic DNA was extracted from pathogenic fungi as described by Zhou et al. (2014). The molecular identities of strains were determined by PCR amplification and sequencing of the nuclear ribosomal DNA ITS region using the following primers: ITS1 (5′-TCCGTAGGTGAACCTGCGG-3′) and ITS4 (5′-TCCTCCGCTTATTGATATGC-3′) (Labbé et al., 2008). Each PCR mixture (50 μL) contained 2.5 μL of genomic DNA, 2.0 μL of each primer (10 μmol/L), 35.5 μL of sterile deionized water, 0.5 μL of Taq PCR Master mix, 5.0 μL of 10× PCR buffer, and 2.5 μL of dNTPs (2.5 mmol/L). The PCR amplification program consisted of 94°C for 5 min, followed by 35 cycles of 94°C for 40 s, 54°C for 1 min and 72°C for 1 min, with a final extension of 72°C for 10 min. The PCR products were sequenced by Sangon Biotech (Shanghai) Co., Ltd., using an ABI-PRISM 3730 automated sequencer (Applied Biosystems, USA). The obtained ITS sequences were compared using BLAST analysis of the NCBI GenBank database.

## Results

### Illumina Sequencing and Community Structure Analysis

**FIGURE 1:**
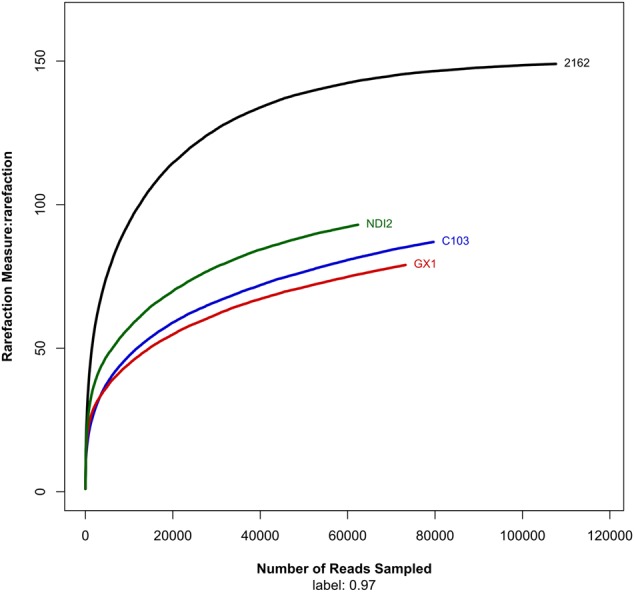
**Rarefaction curves showing the number of unique operational taxonomic units (sharing ≥ 97% sequence identity) per total reads for each sample**.

After denoising and chimera detection, we obtained 62,382 sequences, which were then clustered into 164 OTUs with 97% sequence similarity. The numbers of OTUs detected for the tested soybean varieties “2162,” “C103,” “GX1,” and “ND12” were 146, 85, 71, and 93, respectively (**Table 1**). As indicated by the rarefaction curves shown in **Figure 1**, the number of observed OTUs (Y-axis) increased exponentially with the increasing randomness of ITS1 sequences (X-axis) but then tended to plateau. Although the number of observed OTUs was slightly lower than estimated based on Chao1 and ACE, these values were close to the estimates. The values of the Shannon and Simpson community diversity indexes were 1.74–1.89 and 0.29–0.37, respectively. The coverage data were good, with values higher than 0.9996, indicating sufficient sequencing depth. The above results indicate that the sequencing data are reasonable and that the number of sequences analyzed can sufficiently represent the fungal diversity of all samples.

**Table 1:** Comparison of the estimated operational taxonomic unit (OTU) richness, diversity indexes, and mildew indexes used in this study.

Sample ID	OTU	ACE	Chao1	Shannon	Simpson	Coverage	MIS	MIP
2162	146	150171	149176	1.74	0.38	0.9997	2.50	78.25
C103	85	97146	94153	1.89	0.23	0.9996	1.61	70.19
GX1	71	87132	76124	1.77	0.26	0.9997	41.75	65.67
NDI2	93	100135	97132	1.83	0.28	0.9997	32.90	57.52

**FIGURE 2:**
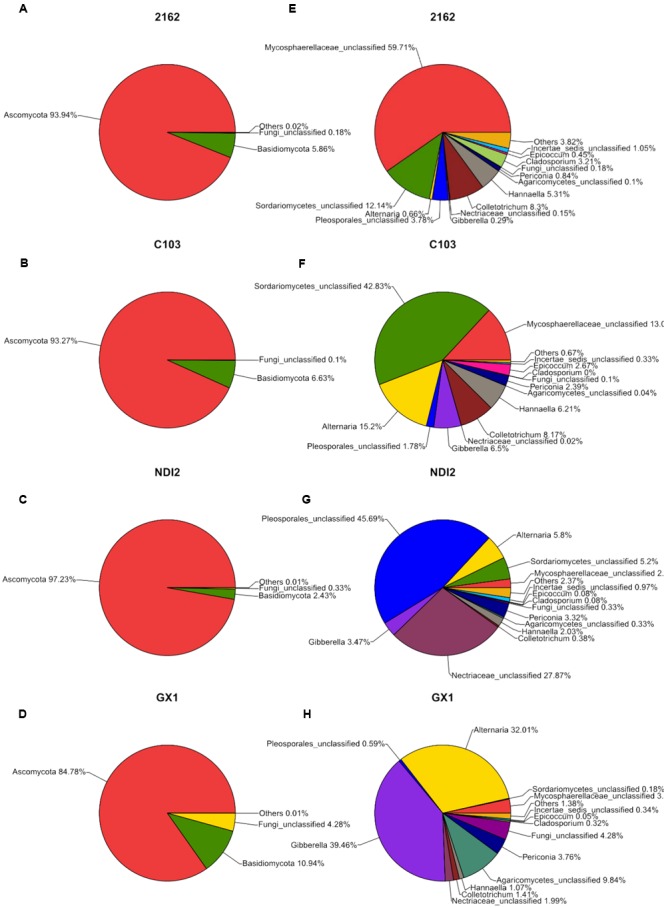
**Relative abundances of fungal phyla (A–D)** and genera **(E–H)** recovered from FM-damaged grains of four soybean varieties by using Illumina high-throughput sequencing.

The fungal community structures at the phylum and genus level in the different soybean varieties are shown in **Figure 2**. As shown in **Figure 2**, the four FM-damaged soybean samples belonged to four phyla: three formally described fungal phyla, Ascomycota, Basidiomycota, and Chytridiomycota, and an unassigned fungal phylum that includes a total of 69 fungal taxa. More than 85% of the total fungal sequences belonged to Ascomycota and Basidiomycota (**Figures 2A–D**); and were mainly distributed among twelve fungal taxa, including Mycosphaerellaceae, Sordariomycetes, and *Alternaria* (**Figures 2E–H**).

### Diversity Analysis among Different Samples

**FIGURE 3:**
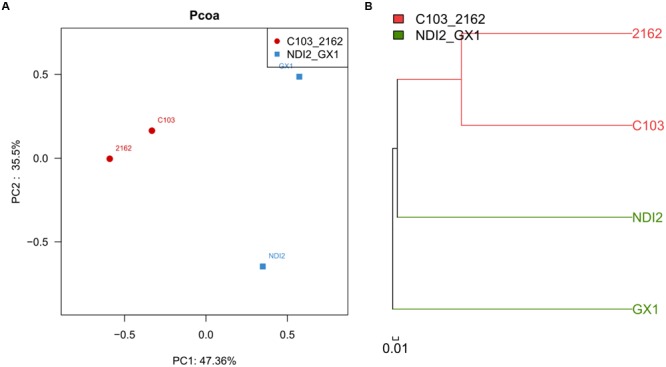
**Principal component analysis (A)** and hierarchical clustering analysis **(B)** of different soybean varieties based on their beta diversity of fungal community composition.

The rDNA ITS sequences of FM infecting four soybean varieties were detected using Illumina high-throughput sequencing. Of these varieties, “2162” and “C103” are black soybeans, whereas “GX1” and “ND12” have yellow seed coats. As shown in **Figure 2**, the relative abundances of fungal genera differed among the varieties. Of the total fungal sequences found on the black soybean “2162,” 59.71% belonged to Mycosphaerellaceae and 12.14% to Sordariomycetes (**Figure 2E**). Similarly, Mycosphaerellaceae (13.0%) and Sordariomycetes (42.83%) were the dominant fungal taxa found on “C103” (**Figure 2F**). In contrast, the dominant fungal communities on the yellow soybean varieties “GX1” and “ND12” differed from those found on the black soybean fruits: Pleosporales (45.69%) and Nectriaceae (27.87%) dominated on “ND12,” and *Gibberella* (39.46%) and *Alternaria* (32.01%) were the dominant fungi on “GX1”. These results were confirmed by PCA (**Figure 3A**) and hierarchical clustering analysis (**Figure 3B**) based on the beta diversity of each fungal community. As shown in **Figure 3A**, based on the first principal component, the four tested soybean varieties were clearly separated, with the black soybeans (“2162” and “C103”) being separated from the yellow soybeans (“GX1” and “ND12”). In addition, “GX1” and “ND12” were separated along the second principal component, whereas “2162” and “C103” were not. A similar result was obtained by using hierarchical clustering analysis, with the two black soybean varieties clustering together but the yellow soybean varieties being grouped into two independent clusters (**Figure 3B**). These results indicate that the fungal diversity among FM-damaged soybean fruits was highly host specific, especially between varieties with different colors of seed coats.

**FIGURE 4:**
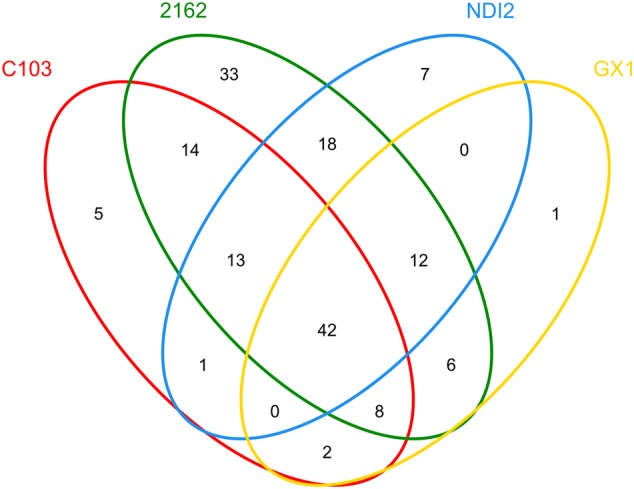
**Venn diagram of OTU distribution among different soybean varieties**.

To further investigate the common relationships among fungal communities of different soybean varieties, the OTU distribution was statistically analyzed, and these results are presented as a Venn diagram in **Figure 4**. For “2162” and “C103,” 77 OTUs (14+13+42+8) were amplified; 54 OTUs (0+42+12+0) were amplified for “GX1” and “ND12,” indicating a more diverse fungal community on black soybean fruits. Additionally, 42 OTUs were amplified from all four different soybean varieties. Further analysis of these 42 OTUs demonstrated that 37 of them belong to Ascomycota, mainly *Alternaria, Gibberella, Hannaella, Colletotrichum, Periconia, Cladosporium, Epicoccum, Boeremia*, and *Fusarium* (**Supplementary Table S1**).

### Relationship between Fungal Diversity and Organ-Specific Mildew Index

**FIGURE 5:**
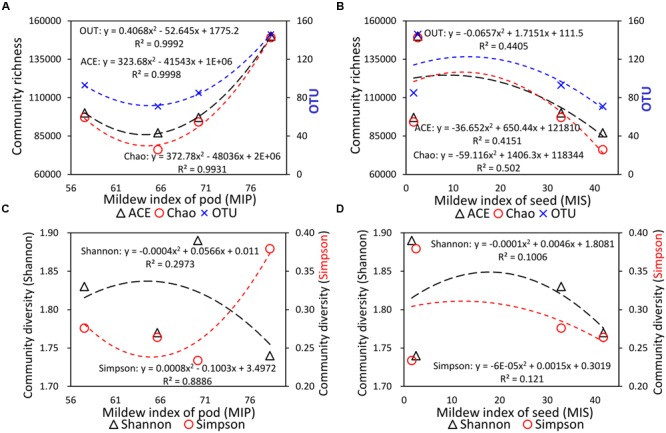
**Quadratic regression analysis between the organ-specific mildew indexes and fungal diversity indexes. (A)** Community richness and MIP; **(B)** community richness and MIS; **(C)** community diversity and MIP; and **(D)** community diversity and MIS.

The current study also determined the organ-specific mildew indexes of the seed (MIS) and pod (MIP) in different soybeans with different field mildew resistances. As shown in **Table 1**, the MIS values for the four tested soybean varieties decreased in the order GX1 > ND12 > 2162 > C103; in contrast, the MIP values decreased in the order 2162 > C103 > GX1 > ND12. To further clarify their relationship, regression analyses were conducted between fungal diversity and organ-specific mildew indexes. The relationship between MIP/MIS values and the indexes of community richness (ACE, Chao1) and OTUs indicated that quadratic models fit the experimental data well (**Figures 5A,B**). The community richness indexes (ACE, Chao1) and OTUs increased as MIP values increased based on a quadratic relationship with high determination coefficients (*R*^2^ > 0.99) (**Figure 5A**). In contrast, these community richness indexes decreased as the MIS values increased based on a quadratic relationship with moderate determination coefficients (*R*^2^ > 0.40) (**Figure 5B**).

Regression analyses were also conducted between organ-specific mildew indexes (MIP, MIS) and the community diversity indexes (Shannon, Simpson) (**Figures 5C,D**). Unlike the community richness indexes, a higher Shannon index means higher community diversity, while a higher Simpson index means lower community diversity (Wang et al., 2012). As shown in **Figure 5C**, with the increase in MIP values, the Shannon community diversity index decreased based on a quadratic relationship (*R*^2^ = 0.2973), and the Simpson community diversity index increased based on a quadratic relationship with high determination coefficients (*R*^2^ = 0.8886). These two regression curves indicated that community diversity decreased as MIP values increased. Furthermore, as shown in **Figure 5D**, with the increase in MIS values, the Shannon and Simpson community diversity indexes decreased based on a quadratic relationship with low determination coefficients (*R*^2^ < 0.15).

### Fungal Isolation and Identification

**FIGURE 6:**
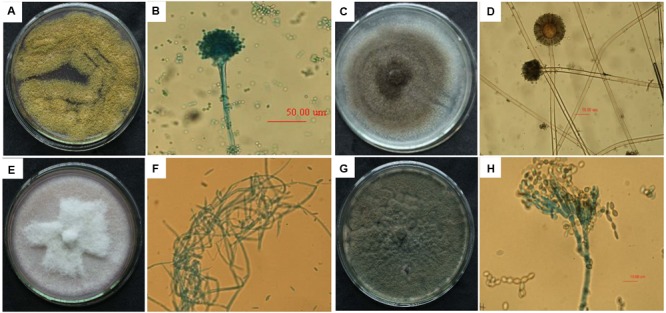
**The colony characteristics and microscopic morphology of *Aspergillus flavus*** (**A**: colony on medium; **B**: conidium terrier and top sac), *A. niger* (**C**: colony on medium; **D**: conidium terrier and top sac), *Fusarium moniliforme* (**E**: colony on medium; **F**: mycelium), and *Penicillium* (**G**: colony on medium; **H**: conidium).

Four single fungal colonies were isolated from the FM-damaged grains of the susceptible soybean variety “ND12”. These colonies were named F1, F2, F3, and F4; sub-cultured; and purified based on morphological characters. The morphological characters of the fungal colonies under study were recorded over 7 days of growth on Martin medium. Microscopic characteristics of conidia or hyphae were measured using an ocular micrometer (**Figure 6**). Based on the comparisons of colony and microscopic characteristics in classical studies, we identified these isolated single fungal colonies (F1, F2, F3, F4) as *Aspergillus flavus* (**Figures 6A,B**), *A. niger* (**Figures 6C,D**)*, Fusarium moniliforme* (**Figures 6E,F**), and *Penicillium* (**Figures 6G,H**), respectively (Ding et al., 2003; Musmade et al., 2013).

To study genetic variation among the above four strains isolated from FM-damaged soybean grains, ITS regions were amplified using rDNA ITS1 and ITS2 primers and then sequenced. The lengths of the sequences among the selected strains ranged from 500 to 650 bp. To search for homologous sequences, the obtained ITS sequences were compared by using BLAST analysis with the NCBI GenBank database. The results of the ITS sequence comparison and molecular identification are shown in **Table 2**, confirming the results of the morphological identification. The F1, F2, F3, and F4 fungal strains were identified as *A. flavus culture-collection MUM:10.220, A. niger isolate PJ-2, Fusarium sp. BAB-4364*, and *P. chrysogenum strain A096*, respectively, with high similarity (**Table 2**).

**Table 2:** Comparative sequence analysis of fungi based on the ITS region and GenBank.

No.	Morphological identification	Molecular identification	Percent similarity	GenBank accession
F1	*Aspergillus flavus*	*Aspergillus flavus culture-collection MUM:10.220*	99%	HQ340108.1
F2	*Aspergillus niger*	*Aspergillus niger isolate PJ-2*	99%	KM460938.1
F3	*Fusarium moniliforme*	*Fusarium sp. BAB-4364*	100%	KM401408.1
F4	*Penicillium*	*Penicillium chrysogenum strain A096*	100%	JQ015265.1

### Pathogenicity Identification

**FIGURE 7:**
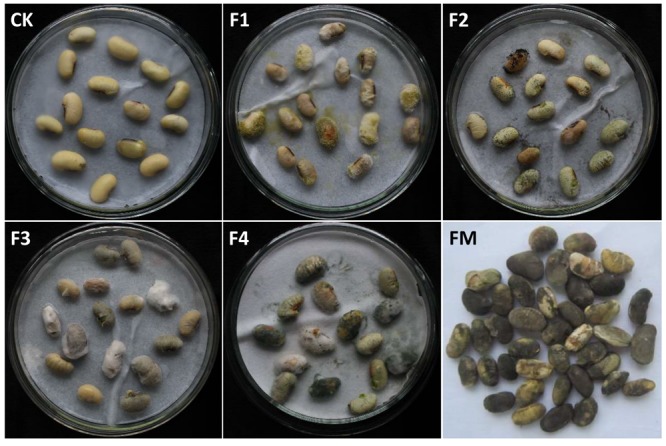
**The mildew phenotype of soybean grains re-inoculated with field-derived fungi. CK**: sterile water; **F1**: *A. flavus*; **F2**: *A. niger*; **F3**: *F. moniliforme*; **F4**: *Penicillium*, **FM**: grains naturally fungus-infected in the field.

The above four isolated candidate pathogenic fungi were re-inoculated onto the surfaces of grains of the FM-susceptible variety “ND12,” and the phenotype was observed after 7 days. As shown in **Figure 7**, the blank control (**Figure 7CK**) soybean grains were simply swollen, without obvious mildew characteristics. In contrast, the other re-inoculated grains showed obvious mold, with a high mildew index (Deng et al., 2015b) above 85. Compared with the pre-harvest naturally fungal-infected soybean seeds in the field (**Figure 7FM**), the grains re-inoculated with *F. moniliforme* (**Figure 7F3**) and *P. chrysogenum* showed a similar mildew phenotype of dark brown color, with a shrunken and cracked surface (**Figure 7F4**). These results indicate that the two fungi identified as F3 and F4 caused the same symptoms as experienced by the field FM-damaged soybean grains. Combining the above information regarding fungal community composition and diversity, *Fusarium* was determined to be the dominant strain on FM-damaged soybean grains; this fungus is widely parasitic to several varieties of soybean. Therefore, we conclude that the pathogenic fungus on the FM-damaged pre-harvested soybean grains was *F. moniliforme*.

## Discussion

### Environmental Factors Determine the Difference between Field and Storage Molds

Grain fungi can be divided into two large categories, field and storage molds, based on the location of the outbreak (Liu et al., 2016). Storage fungi can grow at low water activity, which occurs readily under a high-temperature (25–30°C) and humid environment. In contrast, FM infection typically occurs in the field when the environment is abnormally cold (temperature 13–21°C) and humid (humidity 85–100%). The ability of fungi to germinate, grow and sporulate on grain depends on the availability of water and temperature as well as on the intergranular gas composition (Zhang et al., 2014), and these factors influence grain spoilage by fungi. Different environmental conditions result in different fungal community compositions. For instance, storage mold on *Zea mays* is caused by *A. flavus* and *Penicillium* spp. (Pinto and Fonseca, 2006). To date, there have been many in-depth studies on post-harvest fungal ecology, especially with regards to the impact of fungal growth on stored grain. *F. culmorum, A. ochraceus, Penicillium verrucosum*, and *Fusarium* section *Liseola*, as well as their released fungal toxins, are sources of contaminants in stored cereal grains (Magan et al., 2003). Abnormally cold and wet climatic conditions occasionally result in FM outbreaks in the field before soybean harvest in Southwest China. FM reduces soybean yield and quality and has become the main obstacle to soybean production in Southwest China (Deng et al., 2015b). However, there is scant information to date regarding FM on soybean grains, and the pathogenic fungi have not been elucidated. The current study investigated the fungal diversity among FM-infected soybean grains as well as identifying the pathogenic fungal, filling an important gap in this research.

### Pod Mildew on Soybeans Can Mitigate FM Damage to the Seed

In the current research, we surveyed the organ-specific mildew indexes of four soybean varieties with yellow and black seed coat colors. Our results indicated that the MIP values were higher than the MIS values, which implies that FM primarily occurred on the pod. To confirm this hypothesis, we analyzed the relationship between organ-specific mildew survey data and fungal diversity indexes for different varieties of soybean. The results revealed a close relationship between the FM fungal diversity and the organ-specific mildew indexes of soybean fruit. In particular, there were high determination coefficients (*R*^2^ > 0.99) in the quadratic relationships between the community richness indexes and MIP values. With increases in the MIP, the fungal community richness increased sharply. In stark contrast, the community richness decreased as MIS increased.

Moreover, similar to our previous research, soybean seeds with yellow coats mildewed more easily than did black soybean seeds, indicating that soybean coat color has some type of special relationship with resistance to seed mildew in the field (Deng et al., 2015b). However, this was a speculation without direct evidence to confirm the function of the black seed coat. Our current research provides another possible mechanism for FM resistance. Although black soybean seeds were not more easily infected by FM, their corresponding pods were seriously damaged. In contrast, the opposite results were observed for yellow soybeans, with higher MIS but lower MIP values. It is plausible to suggest that pod mildew on soybeans may mitigate FM damage to the seed in a trade-off between pod and seed mildew.

Our recent research demonstrated a potentially important function of soybean pods in FM resistance at the phytochemical level (Liu et al., 2016). With increases in the contents of infection components, such as proteins, carbohydrates, and fatty acids, the MIP increased and the MIS decreased, demonstrating indirectly that pod mildew can help mitigate the corresponding seed mildew to a certain extent. Seed pods are the outermost barrier between legume seeds and pests and pathogens; our current research provides further evidence of the protective effect of soybean pods in FM resistance.

Additionally, the results of the present study also suggest that the richness and diversity of the FM fungal community were highly host specific among different soybean varieties. Of particular interest was another trade-off between richness and diversity of the FM fungal community. For example, although the FM community richness increased with MIP values in black soybean pods, the FM community diversity in black soybean pods was lower than that in yellow soybean pods. Our current study indicated that the fungal community was more diverse on black soybean seeds, suggesting the existence of antagonistic fungi, although more evidence is needed to confirm this possibility.

### *Fusarium moniliforme* Was the Dominant FM Pathogenic Fungus of Soybean Fruit

In our study, four fungal strains were isolated from the FM-damaged grains of the susceptible soybean variety. The integrated use of multiple methods, including morphological and molecular identification and re-inoculation, confirmed that *F. moniliforme* was the dominant FM pathogenic fungus among our samples, and we identified the strain as *Fusarium sp. BAB-4364*. Various *Fusarium* species not only cause seed rot and pre-emergence and post-emergence seedling damage but also result in wilting during the growth of numerous species of plants, including various cereals, vegetables, pulses, and oilseed crops (Muzahid et al., 2010). *F. moniliforme* is one of the most important pathogens causing several damaging crop diseases and producing enormous losses; of particular note are stalk rot and ear rot disease of maize, which have now achieved great importance in maize-growing areas (Musmade et al., 2013). Indeed, *F. moniliforme* produces mycotoxins, which are potential health hazards to humans and animals that frequently consume maize and maize products (Abeni et al., 2014). Species of *Fusarium* known to be seed-borne pathogens cause enormous losses to crops worldwide, and these fungi are frequently present on plant seeds; some are serious pathogens, whereas others are weakly pathogenic and still others merely saprophytes (Muzahid et al., 2010). Some species of *Fusarium* are seed-borne pathogens of soybean, especially *F. moniliforme*, which is responsible for wilt, root rot and foot rot (Muzahid et al., 2010); other species include *F. anguioides, F. equiseti, F. fusarioides, F. moniliforme, and F. poae* (Muzahid et al., 2010). However, reports of soybean fruit rot resulting from *Fusarium* are rare.

Additionally, *Fusarium* has been described as a latent pathogen, one that can function in both asymptomatic and symptomatic infections (Impullitti and Malvick, 2014). The slightly FM-damaged soybean fruit clearly displayed mildew symptoms on the pod surface, while the seed appeared healthy. However, during asymptomatic infection, the hyphae of *Fusarium* may already colonize intercellular spaces. When soybean plants suffer serious FM damage, which is a symptomatic infection, the hyphae of *Fusarium* may colonize plant tissues both inter- and intracellularly, and the biomass of the infected site will be reduced. In this situation, the seriously FM-damaged soybean seed exhibited a mildew phenotype with a dark brown color and a shrunken and cracked surface. The same symptoms were observed in soybean seeds re-inoculated with *F. moniliforme* (Deng et al., 2015a). In conclusion, the seriously FM-damaged soybean pod was more likely to be a symptomatic infection, while the slightly FM-damaged soybean seed was more likely to be symptomless endophytic colonization.

## Conclusion

To the best of our knowledge, this is the first exploration of the diversity of FM fungal communities on soybean grains using Illumina high-throughput sequencing. Our results based on morphological and molecular identifications implicate *F. moniliforme* as the dominant FM pathogen. Additionally, the fungal community composition varied among specific organs and among different soybean varieties. This host specificity of FM fungal diversity with regard to different varieties as well as different tissues may help to promote FM resistance in soybean.

## Author Contributions

Study idea and design: JL, X-lC, and W-yY. Field and lab work: J-cD, NH, W-gL, FY, and C-qY. Data analysis: JZ, KS, and T-wY. Paper concept and writing: JL, J-cD, and X-cW. All authors discussed the results and commented on the manuscript at all stages.

## Conflict of Interest Statement

The authors declare that the research was conducted in the absence of any commercial or financial relationships that could be construed as a potential conflict of interest.

## Footnotes

1 http://drive5.com/uparse/

## Supplementary Material

TABLE S1 **Taxonomic information of 42 common OTUs of fungal taxa detected on four soybean varieties**.
